# Supplementary material for: Compartmentalized metabolism supports midgestation mammalian development
Source: Nature. 2022 Apr 6;604(7905):349–53. doi: 10.1038/s41586-022-04557-9 (PMC9007737; doi:10.1038/s41586-022-04557-9)
Supplement: Supplementary file 1 — A list of abbreviations. [file 41586_2022_4557_MOESM1_ESM.pdf]

nature portfolio

https://doi.org/10.1038/s41586-022-04557-9

Supplementary information

Abbreviation	Full Name	Abbreviation	Full Name
[U-13C]glucose	uniformily 13C-labeled glucose (m+6)	H&E	Hematoxylin and eosin
[U-13C]glutamine	uniformily 13C-labeled glutamine (m+5)	Hand1	Heart- and neural crest derivatives-expressed protein 1
2OADH	2-oxoadipate dehydrogenase	HIF1α	Hypoxia inducible factor 1 alpha
adenylosuc	Adenylosuccinate	IMP	inosine monophosphate
α-kg	alpha ketoglutarate	Itg4a	Integrin Subunit Alpha 4
AKGDH	alpha ketoglutarate dehydrogenase	KIC	alpha-ketoisocaproate
AMP	adenosine monophosphate	KIV	alpha-ketoisovalerate
Arnt	Aryl Hydrocarbon Receptor Nuclear Translocator	KMV	alpha-keto-beta-methylvalerate
Asp	aspartate	Lac	lactate
BCKDH	branched chain amino acid dehydrogenase	LIPT1	Lipoyltransferase I
Cdx2	caudal type homeobox 2	Lys	lysine
Aco	cis-aconitate	Mal	malate
Cit	citrate	MEP	myeloid/erythroid progenitor
CMP	cytodine monophosphate	MSOA	metabolic set overrepresentation analysis
Cox	cytochorme c oxidase subunits	Nduf	NADH:Ubiquinone Oxidoreductase Subunit
C-section	cesarean section	P	placenta
Cyc1	Cytochrome c1	PC	pyruvate carboxylase
Cycs	cytochrome c somatic	PDH	pyruvate dehydrogenase
DHAP	dihydroxyacetone phosphate	PE	PECAM/Endomucin
Dlx3	Distalless 3	Pparg	peroxisome proliferator-activated receptor γ
E	embryo	Pyr	pyruvate
Egfr	Epidermal growth factor receptor	R5P	ribose 5-phosphate
Eomes	Eomesodermin	RBC	red blood cell
Ery	erythrocyte	RiB5p	Ribulose 5 phosphate
Esx1	ESX Homeobox 1	SDHb	succinate dehydrogenase subunit b
ETC	electron transport chain	Suc	succinate
Ets2	ETS Proto-Oncogene 2	TCA	tricarboxylic acid
F6P	fructose 6-phosphate	UMP	uridine monophosphate
FC	fold change	Vcam	Vascular cell adhesion molecule
G6P	Glucose 6-phosphate	WBC	white blood cells
Gcm1	glial cells missing 1	X5P	Xyulose 5 phosphate
gd	gestational day		
Gjb2	Gap Junction Protein, Beta 2		
Gln	glutamine		
Glu	glutamate		
GMP	guanosine monophosphate		
guanidine ace	guanidine acetate		
