# Supplementary material for: Compartmentalized metabolism supports midgestation mammalian development
Source: Nature. 2022 Apr 6;604(7905):349–53. doi: 10.1038/s41586-022-04557-9 (PMC9007737; doi:10.1038/s41586-022-04557-9)
Supplement: Supplementary file 2 — Reporting Summary [file 41586_2022_4557_MOESM2_ESM.pdf]

nature portfolio

Corresponding author(s): Ralph DeBerardinis

Last updated by author(s): 2021/12/08

Reporting Summary

Nature Portfolio wishes to improve the reproducibility of the work that we publish. This form provides structure for consistency and transparency in reporting. For further information on Nature Portfolio policies, see our Editorial Policies and the Editorial Policy Checklist.

Statistics

For all statistical analyses, confirm that the following items are present in the figure legend, table legend, main text, or Methods section.

n/a	Confirmed	
☐	☒	The exact sample size (*n*) for each experimental group/condition, given as a discrete number and unit of measurement
☐	☒	A statement on whether measurements were taken from distinct samples or whether the same sample was measured repeatedly
☐	☒	The statistical test(s) used AND whether they are one- or two-sided<br>*Only common tests should be described solely by name; describe more complex techniques in the Methods section.*
☐	☒	A description of all covariates tested
☐	☒	A description of any assumptions or corrections, such as tests of normality and adjustment for multiple comparisons
☐	☒	A full description of the statistical parameters including central tendency (e.g. means) or other basic estimates (e.g. regression coefficient) AND variation (e.g. standard deviation) or associated estimates of uncertainty (e.g. confidence intervals)
☐	☒	For null hypothesis testing, the test statistic (e.g. *F*, *t*, *r*) with confidence intervals, effect sizes, degrees of freedom and *P* value noted<br>*Give P values as exact values whenever suitable.*
☒	☐	For Bayesian analysis, information on the choice of priors and Markov chain Monte Carlo settings
☒	☐	For hierarchical and complex designs, identification of the appropriate level for tests and full reporting of outcomes
☒	☐	Estimates of effect sizes (e.g. Cohen's *d*, Pearson's *r*), indicating how they were calculated

Our web collection on statistics for biologists contains articles on many of the points above.

Software and code

Policy information about availability of computer code

Data collection	Flow cytometry data were collected using LSRFortessa cell analyser (Becton Dickinson). GC-MS data were collected using Agilent ChemStation E02.02.1431, LC-MS/MS data were collected using SCIEX Analyst v1.6.3 and Thermo Scientific XCalibur 4.1.50. Quantitative PCR data were collected using a Bio-Rad CFX384 Touch Real-Time PCR Detection machine. Whole mount IF images were obtained using a LSM700 Ziess confocal microscope. Whole heart and H&E stained slides were imaged using a Ziess Images M2 with an Axiocam 506 mono camera attached.
Data analysis	GraphPad Prism V9.0.1 or R 4.0.2 with the stats, fBasics, car, and nparLD packages, Flow cytometry data analysis using BD FACSDiva 8.0, and FlowJo V10 (Treestar). GC-MS data analysis using Agilent ChemStation E02.02.1431. LC-MS/MS data analysis using SCIEX Multiquant v2.1.1 and Thermo Scientific Trace Finder 5.1, IF, whole heart and H&E images were analyzed with the Carl Zeiss ZEN 2011 software. Statistical analysis for generation of PCA plots, heatmaps, differential abundances and metabolic set overrepresentation analysis were performed using MetaboAnalyst 5.0 (https://www.metaboanalyst.ca). For 13C studies, observed distributions of mass isotopologues were corrected for natural isotope abundances using a customized R script, which can be found at the GitHub repository (https://github.com/wencgu/nac). The script was written by adapting the AccuCor algorithm v0.2.4. Gene expression data obtained using the GEOquery package v2.62. (DOI:10.18129/B9.bioc.GEOquery) from BioConductor release 3.14 (https://www.bioconductor.org/). Further data analysis was performed using the metaboAnalyst_KEGG R package (https://github.com/xia-lab/MetaboAnalystR) and the HomoloGene database ((https://www.ncbi.nlm.nih.gov/homologene).

For manuscripts utilizing custom algorithms or software that are central to the research but not yet described in published literature, software must be made available to editors and reviewers. We strongly encourage code deposition in a community repository (e.g. GitHub). See the Nature Portfolio guidelines for submitting code & software for further information.

Data

Policy information about availability of data

All manuscripts must include a data availability statement. This statement should provide the following information, where applicable:

- Accession codes, unique identifiers, or web links for publicly available datasets
- A description of any restrictions on data availability
- For clinical datasets or third party data, please ensure that the statement adheres to our policy

Data supporting the findings of this study are available within the article and its Supplementary Information files or from the corresponding author on request. Source Data for Figures 1-4 and Extended Data Figures 1-6 are provided with the paper as supplementary tables. RNAseq data for fetal tissues during midgestation are available from the ENCODE project Mouse Development Matrix (https://www.encodeproject.org/mouse-development-matrix). We downloaded the tsv files from the polyA plus RNAseq assay with the following identifiers: ENCFF262TPS (E11.5 liver -1), ENCFF414APX (E11.5 liver-2), ENCFF173NFQ (E12.5 liver-1), ENCFF144DHB (E12.5 liver-2), ENCFF971KKK (E13.5 liver-1), ENCFF042DVY (E13.5 liver-2), ENCFF770SOB (E10.5 heart-1), ENCFF351QKG (E10.5 heart-2), ENCFF159DWP (E11.5 heart-1), ENCFF168UJM (E11.5 heart-2), ENCFF484QWQ (E12.5 heart-1), ENCFF329HOZ (E12.5 heart-2), ENCFF148BEQ (E13.5 heart-1), ENCFF836QQS (E13.5 heart-2), ENCFF145PTV(E10.5 forebrain-1), ENCFF476ADM (E10.5 forebrain-2), ENCFF606UHO (E11.5 forebrain-1), ENCFF434CSI (E11.5 forebrain-2), ENCFF928MQD (E12.5 forebrain-1), ENCFF046RSQ (E12.5 forebrain-2), ENCFF960KJV (E13.5 forebrain-1), ENCFF356CTG (E13.5 forebrain-2). Placenta RNA transcript abundance was obtained from Soncin et. al. (GSE100053). Expression data were filtered based on known metabolic genes47-49 and human-mouse gene mapping was based on the HomoloGene database (https://www.ncbi.nlm.nih.gov/homologene).

Field-specific reporting

Please select the one below that is the best fit for your research. If you are not sure, read the appropriate sections before making your selection.

☒ Life sciences ☐ Behavioural & social sciences ☐ Ecological, evolutionary & environmental sciences

For a reference copy of the document with all sections, see nature.com/documents/nr-reporting-summary-flat.pdf

Life sciences study design

All studies must disclose on these points even when the disclosure is negative.

Sample size	Samples sizes were not pre-determined based on statistical power calculations but were based on our experience with these assays. For most experiments, the minimum number of mice was 3, with some exceptions where the embryo/placenta numbers were n≥10. For metabolomics and isotope tracing studies in mice, our previous publications (PMID: 31853067 PMCID: PMC6930341, PMID: 28985563 PMCID: PMC5684706) have determined that 3-5 mice is a sufficient sample size to obtain statistical power. Because our statistics was performed on placenta and embryo numbers that exceed the number of mice in the study, we determined that using 3 mice would be sufficient as biological replicates unless the number of placenta/embryos obtained from 2 mice was equivalent to experiments where 3 mice were used. This number was typically n>10.
Data exclusions	No data were excluded; however, sometimes the small sample size was below the threshold for metabolomic analysis. In those instances, data that could be obtained from maternal blood or other tissues was used. These samples were not used during direct comparisons of embryo relative to its own placenta if one of the samples was absent. As well, any conceptus that was observed to contain hemorrhage or necrotic tissue was deemed "non-viable" and not used for any additional studies.
Replication	The experimental findings were reproduced in multiple independent experiments. The number of independent experiments and biological replicates for each data panel is indicated in the figure panel itself or in the figure legends, and in the source data files. Data shown in the figures represent the aggregate of all independent experiments in most cases. Data shown in a minority of panels are from a representative experiment (e.g. for histology) and in those cases the number of independent experiments that reproduced the finding is also indicated in the figure legends. For patient data, we show cell counts that were included in the single patients chart over her lifetime.
Randomization	No formal randomization techniques were used; however, samples were allocated randomly to experiments and processed in an arbitrary order.
Blinding	During flow cytometry, isotope tracing, metabolomics, qPCR, tissue weights, somite counts and histology experiments, the data were analyzed in a manner blinded to sample genotype. A.S. collected the samples and then passed them to A.T. for flow cytometry, or to I.M-M. and M.A.C. for histology and immunofluorescence, and A.T. for qPCR. A.S. processed samples for mass spectrometry and analyzed data. After the patterns had been analyzed in each of these experiments, D.D. provided the genotype information so results could be interpreted. For experiments in wild-type mice, no blinding was performed on placentas versus embryos because A.S. performed these experiments and analyzed the data. For gene expression studies from publicly available datasets, no blinding was performed.

Reporting for specific materials, systems and methods

We require information from authors about some types of materials, experimental systems and methods used in many studies. Here, indicate whether each material, system or method listed is relevant to your study. If you are not sure if a list item applies to your research, read the appropriate section before selecting a response.

Materials & experimental systems

n/a	Involved in the study
☐	☒ Antibodies
☒	☐ Eukaryotic cell lines
☒	☐ Palaeontology and archaeology
☐	☒ Animals and other organisms
☐	☒ Human research participants
☐	☒ Clinical data
☒	☐ Dual use research of concern

Methods

n/a	Involved in the study
☒	☐ ChIP-seq
☐	☒ Flow cytometry
☒	☐ MRI-based neuroimaging

Antibodies

Antibodies used

The following antibodies have been used in this study:

Anti-Mouse Ter119 APC
clone: TER-119
REF 20-5921-U100
LOT C5921040320203,
TONBO
1:100 Flow

Anti-Mouse CD71 FITC
clone R17217 (R17 217.1.4)
LOT 2213055
REF 2213055
Biolegend
1:100 Flow

Anti-Mouse CD41 PE/Cy7
clone: MWReg30
cat 133916
LOT B27086
Biolegend
1:100 Flow

Anti-Mouse CD117 (c-KIt) APC-eFluor 780
clone: 2B8
Ref: 47-1171-82
LOT 2261910
Invitrogen
1:100 Flow

Rat-anti-PECAM
clone: MEC 13.3 (RUO)
BD, Biosciences,
cat #553370
1:100 Whole mount IF

Rat-anti-Endomucin
clone: V.7C7
Santa Cruz, sc-65495
1:100 Whole mount IF

Rabbit-anti-Connexin 40
Alpha Diagnostics International, cat#CX-40A
1:100 Whole mount IF

donkey-anti-rat 488
Invitrogen, cat #A21208
1:250 secondary Whole mount IF

donkey-anti-rabbit 555
Invitrogen, cat# A31572
1:250 secondary Whole mount IF

Validation

All antibodies are commercially available and have been validated in previously published studies

Anti-Mouse Ter119 APC:
Egusquiza RJ, Ambrosio ME, Wang SG, Kay KM, Zhang C, Lehmler HJ, Blumberg B. Evaluating the Role of the Steroid and Xenobiotic

Receptor (SXR/PXR) in PCB-153 Metabolism and Protection against Associated Adverse Effects during Perinatal and Chronic Exposure in Mice. Environ Health Perspect. 2020 Apr;128(4):47011. doi: 10.1289/EHP6262. Epub 2020 Apr 30.

Anti-Mouse CD71 FITC:
Tsai S, Clemente-Casares X, Zhou AC, Lei H, Ahn JJ, Chan YT, Choi O, Luck H, Woo M, Dunn SE, Engleman EG, Watts TH, Winer S, Winer DA. Insulin Receptor-Mediated Stimulation Boosts T Cell Immunity during Inflammation and Infection. Cell Metab. 2018 Dec 4;28(6):922-934.e4. doi: 10.1016/j.cmet.2018.08.003. Epub 2018 Aug 30. PMID: 30174303.

Anti-Mouse CD41 PE/Cy7:
Gentek R, Ghigo C, Hoeffel G, Bulle MJ, Msallam R, Gautier G, Launay P, Chen J, Ginhoux F, Bajénoff M. Hemogenic Endothelial Fate Mapping Reveals Dual Developmental Origin of Mast Cells. Immunity. 2018 Jun 19;48(6):1160-1171.e5. doi: 10.1016/j.immuni.2018.04.025. Epub 2018 May 29. PMID: 29858009.

Anti-Mouse CD117 (c-KIt) APC-eFluor 780:
Di Genua C, Valletta S, Buono M, Stoilova B, Sweeney C, Rodriguez-Meira A, Grover A, Drissen R, Meng Y, Beveridge R, Aboukhalil Z, Karamitros D, Belderbos ME, Bystrykh L, Thongjuea S, Vyas P, Nerlov C. C/EBPα and GATA-2 Mutations Induce Bilineage Acute Erythroid Leukemia through Transformation of a Neomorphic Neutrophil-Erythroid Progenitor. Cancer Cell. 2020 May 11;37(5):690-704.e8. doi: 10.1016/j.ccell.2020.03.022. Epub 2020 Apr 23. PMID: 32330454; PMCID: PMC7218711.

Rat-anti-PECAM:
Baldwin HS, Shen HM, Yan HC, et al. Platelet endothelial cell adhesion molecule-1 (PECAM-1/CD31): alternatively spliced, functionally distinct isoforms expressed during mammalian cardiovascular development. Development. 1994; 120(9):2539-2953. (Clone-specific: Blocking).

Rat-anti-Endomucin:
Wang, C.|Ying, J.|Nie, X.|Zhou, T.|Xiao, D.|Swarnkar, G.|Abu-Amer, Y.|Guan, J.|Shen, J.| et al. 2021. Bone Res. 9: 29.PMID: # 34099632

Rabbit-anti-Connexin 40
Shekhar A, Lin X, Liu F, Zhang J, Mo H, Bastarache L, et al. Transcription factor ETV1 is essential for rapid conduction in the heart. J Clin Invest. 2016;126:4444-4459

Animals and other organisms

Policy information about studies involving animals; ARRIVE guidelines recommended for reporting animal research

Laboratory animals

All mice were housed in a pathogen free environment (Temperature 68-79F, Humidity: 30-70%) with a 12:12 light/dark cycle and fed chow diet ad libitum. Wild-type C57/BL6 females and males were obtained either from Jackson Labs or from the UT-Soutwestern Breeding Core Facility. Males were only used to initiate pregnancies and were between 3-6 months old. Females were 8-12 weeks old and naively pregnant.

LIPT1 N44S mice were generated at the Children's Research Institute (CRI) Mouse Genome Engineering Core as previously described (Ni M, et. al. Functional Assessment of Lipoyltransferase-1 Deficiency in Cells, Mice, and Humans. Cell Rep. 2019 Apr 30;27(5):1376-1386.e6. doi: 10.1016/j.celrep.2019.04.005. PMID: 31042466; PMCID: PMC7351313.) These mice were back crossed to C57/BL6 mice for at least 10 generations and maintained by breeding either as heterozygous breeding pairs or crossed to C57/BL6. Gender specific breeding was not used. Female LIPT1 WT/N44S pregnant mice (8-15 week) were used for experiments.

Wild animals

no wild animals were used in these studies.

Field-collected samples

no field-collected samples were used in these studies

Ethics oversight

All procedures were approved by the UT Southwestern Animal Care and Use Committee (IACUC) in accordance with the Guide for the Care and Use of Laboratory Animals.

Note that full information on the approval of the study protocol must also be provided in the manuscript.

Human research participants

Policy information about studies involving human research participants

Population characteristics

This manuscript describes a single female patient diagnosed with LIPT1 deficiency (genotype: LIPT1: c.875C > G (p.S292X),c.131A > G (p.N44S)) that has been followed since shortly after birth to a current age of 16.

Recruitment

This patient was recruited as part of a larger study (NCT02650622).

Ethics oversight

All subjects were enrolled in the study (NCT02650622) approved by the Institutional Review Board (IRB) at University of Texas Southwestern Medical Center (UTSW). Informed consent was obtained from all patients and their families.

Note that full information on the approval of the study protocol must also be provided in the manuscript.

Clinical data

Policy information about clinical studies

All manuscripts should comply with the ICMJE guidelines for publication of clinical research and a completed CONSORT checklist must be included with all submissions.

Clinical trial registration	NCT02650622
Study protocol	https://clinicaltrials.gov/ct2/show/NCT02650622
Data collection	n/a
Outcomes	n/a

Flow Cytometry

Plots

Confirm that:

☒ The axis labels state the marker and fluorochrome used (e.g. CD4-FITC).

☒ The axis scales are clearly visible. Include numbers along axes only for bottom left plot of group (a 'group' is an analysis of identical markers).

☒ All plots are contour plots with outliers or pseudocolor plots.

☒ A numerical value for number of cells or percentage (with statistics) is provided.

Methodology

Sample preparation	Whole embryos were dissected and dissociated in cold-PBS using a disposable pestle (VWR). To obtain a single-cell suspension, cells were filtered through a 40 um cell strainer and then stained with the appropriate antibodies.
Instrument	BD Fortessa (for analysis)
Software	BD FACSDiva 8.0, FlowJo V10
Cell population abundance	30,000 cells were sorted from each embryo. CD71-/TER119- cells were 10% of the total population, CD71+/TER119+ (Erythrocytes) cells were 0.5-1% of the total population. CD71-/TER119-/CD41+ (MEPs) cells were 0.2-0.7% of the population.
Gating strategy	To eliminate dead cells from analyses, cells were stained with 4',6-diamidino-2-phenylindole (DAPI). Mouse erythrocytes were identified as being positive for endothelial markers Ter119 and CD71. Megakaryocyte and erythroid progenitor cells were identified as negative for TER119, CD71, and CD117 (c-KIT) but positive for CD41.

☒ Tick this box to confirm that a figure exemplifying the gating strategy is provided in the Supplementary Information.
